# Characteristics of 100 consecutive patients with COVID-19 referred to consultation-liaison psychiatry services in Qatar: A comparison of patients with delirium versus other psychiatric diagnoses

**DOI:** 10.5339/qmj.2022.28

**Published:** 2022-06-17

**Authors:** Yousaf Iqbal, Majid Alabdulla, Rajeev Kumar, Javed Latoo, Sultan Albrahim, Ovais Wadoo, Ovais Haddad

**Affiliations:** ^1^Psychiatry Hospital, Hamad Medical Corporation, Doha, Qatar E-mail: yiqbal@hamad.qa; ^2^College of Medicine, Qatar University, Qatar; ^3^Division of Psychology and Mental Health, University of Manchester, UK

**Keywords:** COVID-19, SARS-Cov2, delirium, consultation–liaison psychiatry, hypoxia, inflammatory markers, mortality

## Abstract

Introduction: Coronavirus disease 2019 (COVID-19) can present with various neuropsychiatric manifestations. This study reports on patients with COVID-19 who were referred to the consultation–liaison (CL) psychiatry services in Qatar and compares the clinical and sociodemographic characteristics of those diagnosed with delirium versus other psychiatric diagnoses. Methods: This is a retrospective review of the first 100 consecutive patients with COVID-19 who were referred to the CL services. Results: Within the total cohort (n=100), most patients (92%) were male, and the mean age was 46 years. About 27% of patients had asymptomatic COVID-19, 35% had a past psychiatric history, and 48% reported pandemic related psychosocial stress. Delirium was the most common psychiatric diagnosis (n=29), followed by acute stress reaction/adjustment disorder, depression, mania, anxiety, non-affective psychosis, and dementia. Among patients with delirium, agitation was the most common symptom (76%), 86% were treated with psychotropic medications, and 17% died. Higher age, longer hospital stays, lower oxygen saturation, lower lymphocytic count, and higher C-reactive protein (CRP) values were significantly associated with delirium versus other psychiatric diagnoses. Higher age and lower oxygen saturations predicted delirium.Conclusion: Delirium was associated with a range of clinical variables and had significant mortality, despite the relatively young age of the patients. COVID-19 should be considered in patients presenting with delirium. Finally, early identification and management of delirium should be integral to COVID-19 protocols.

## Introduction

Patients with Coronavirus disease 2019 (COVID-19) can present with a range of neuropsychiatric disorders, including delirium^1–6^. Delirium in COVID-19 can reflect multiple factors, including inflammation, endothelial damage, increased oxidative stress, hypoxemia, iatrogenic factors, and potentially direct infection of the central nervous system by SARS-CoV-2^7,8^. In fact, delirium has been reported as the presenting and only feature of COVID-19^7,9^. A recent systematic review and meta-analysis of delirium in COVID-19 patients identified its prevalence and incidence rates as 24.3% and 32.4%, respectively^10^. Other studies have also demonstrated that delirium associated with COVID-19 is more prevalent in the elderly, who are also more prone to developing severe COVID-19^11^. Delirium in COVID-19 patients has been associated with in-hospital death, increased length of hospital stay, intensive care admission, and ventilator utilization; it has also been reported to be a predictive factor of inhospital death and adverse outcomes in hospitals^12^.

In this study, we conducted a retrospective review of the electronic patient records for the first 100 consecutive patients with COVID-19 referred to the consultation–liaison (CL) psychiatry service in Doha after Qatar recorded its first COVID-19 case (February 2020)^13^. The aim was to better understand the psychiatric morbidity associated with acute COVID-19 among patients referred to a CL service.

## Methodology

We have previously reported on the first 50 patients seen in this cohort^14^. In the current report, we describe the full cohort of 100 patients and compare the characteristics of those diagnosed with delirium versus other psychiatric diagnoses. During the pandemic, the Qatar CL service operated across three hospitals in Doha managed by the Hamad Medical Corporation (HMC). The inclusion criteria for the study were individuals aged 18 years and over and a positive real-time polymerase chain reaction test for Severe Acute Respiratory Syndrome Coronavirus-2 (SARS-CoV-2) during the index admission. The 100 cases presented between February 28^th^ and August 3^rd^, 2020. Psychiatric diagnoses were those recorded by the CL team in the patient records and reflected the team's clinical judgment. The study was approved by the HMC Institutional Review Board (MRC-05–072).

## Results

Table 1 shows the characteristics of the 100 patients. Most (92%) were male, mean age was 46 years, 27% had asymptomatic COVID-19 (i.e., no characteristic physical symptoms of COVID-19), and 35% had a past psychiatric history. Significant recent psychosocial stress, which is largely related to the pandemic, was recorded in the notes of 48 patients. A psychiatric diagnosis was made by the CL team in all but one case. Delirium was the most common psychiatric diagnosis (n=29), followed by acute stress reaction or adjustment disorder (n=25), depression (n=16), mania (n=15), anxiety (n=14), non-affective psychosis (n=13), and dementia (n=6). Nearly half of the delirium cases (n=12/29) had a medical comorbidity (i.e., a current medical condition other than COVID-19). Moreover, sleep disturbance was the most common psychiatric symptom (n=65, 65%), followed by anxiety (n=52, 52%) among all 100 patients. Recent self-harm thoughts or attempts were present in 24 (24%) of all patients with COVID-19 referred to the CL team. The most common psychiatric symptoms in the delirium cohort were agitation (n 22, 76%), impaired memory (n=19, 66%), disorientation (n=18, 62%), sleep-wake disturbance (n=11, 38%), and confusion (n=11, 38%). Recent self-harm thoughts or attempts were present in 2 patients in the delirium cohort. Psychotropic medications were prescribed to 25 out of the 29 (86%) patients with delirium during their admission. The most common medications were haloperidol (n=17), benzodiazepines (n=10), and quetiapine (n=9), and low doses were used in all cases. All patients with delirium were offered non-pharmacological management strategies including advice to the general medical team to assist with orientation, reassurance, and effective communication. About 17% (n=5) of patients with delirium died during the index admission, while 3% (n=2) in the non-delirium group died; both patients had metastatic cancer. The 5 patients with delirium who died all suffered from COVID-19 severe pneumonia/critical disease.

A univariate comparison of the characteristics of patients with and without delirium showed older age, a prolonged hospital stay, lower oxygen saturation, higher C-reactive protein (CRP) value, lower lymphocyte count, and a range of treatment variables (use of steroids, antivirals/antibiotics and chloroquine/hydroxychloroquine) as factors that were significantly associated with a diagnosis of delirium (Table 2). Binary logistic regression using a stepwise method, which was employed to control for significant variables in the univariate analysis, revealed higher age and lower oxygen saturations that had statistically significant links with a delirium diagnosis (Table 3

## Discussion

Our data revealed that delirium was the most common psychiatric diagnosis seen by the CL team in COVID-19 patients in the early months of the pandemic. Our cohort of patients with delirium is younger than that reported in other studies of delirium related to COVID-19^11,15,16^. This is consistent with Qatar's relatively young population (median age of 33.7 years)^17^.The most prevalent symptoms of delirium in our case series included sleep–wake disturbances and agitation. Hyperactive delirium has been reported to be more common in younger adults with COVID-19, whereas the hypoactive form appears more common in elderly patients^18^. Furthermore, patients with hyperactive delirium may be more likely to be referred to psychiatric services than those with hypoactive delirium.

In our study, over a quarter of COVID-19 patients who were referred to the CL team were physically asymptomatic (i.e., they had no characteristic physical symptoms of COVID-19). These included patients who were transferred from quarantine centers, from psychiatric hospitals, or were admitted following screenings at emergency departments where they first presented with psychiatric symptoms. Nearly one quarter of the patients in the full cohort of 100 patients reported self-harm thoughts or attempts. The diverse psychiatric symptoms seen in the full cohort could reflect the effects of psychosocial stressors, such as quarantine, social distancing, and financial difficulties, the physical effects of SARS-coV2 including potential direct neurotoxic effects and iatrogenic factors^19^.

Our finding of an association between delirium and raised CRP level suggests that inflammation may be involved in the pathophysiology of COVID-19-associated delirium. Furthermore, raised CRP level has also been found to be independently associated with acute renal failure in COVID-19 patients^20^. Higher age and lowest oxygen saturation were significantly associated with delirium, as previously reported^11,21^.

In the non-delirium group, patients presented with a range of psychiatric diagnoses, including acute stress reaction/adjustment disorder, depression, anxiety disorders, non-affective psychosis, and first onset mania. Sleep disturbance was the most common psychiatric symptom among all patients with COVID-19 referred to the CL service, and other studies have also highlighted its high prevalence^22^. This underscores the importance of recognition and the management of sleep disturbances in clinical practice. Furthermore, nearly two-thirds of patients in the non-delirium group had no past psychiatric history, thus supporting the view that COVID-19 was likely to be a significant etiological factor in the appearance of these disorders.

The first limitation of this study is that it only assessed patients referred to the HMC CL services. There are likely to be COVID-19 patients who had delirium, especially during intensive care unit (ICU) admissions, who were not referred to CL services because their psychiatric symptoms may not have posed significant management issues. Second, this was an observational study that utilized secondary data. Thus, it is dependent on the quality of the information recorded in the notes.

An important finding from our study is that delirium can occur in relatively young and otherwise healthy patients with COVID-19. Moreover, delirium is not invariably associated with severe COVID-19 illness but can present in people who are physically asymptomatic or have mild physical symptoms of COVID-19. Therefore, it is important to consider a diagnosis of COVID-19 in patients presenting with delirium. In our results, we recorded a 17% (n=5/29) mortality in the delirium cohort despite the relatively young mean age of these patients (59.4 years).

## Conclusion

Del irium was the most common psychiatric disorder seen in patients with acute COVID-19 who were referred to CL services in Qatar—a country with a young, predominantly migrant population. Furthermore, the data showed that delirium was not invariably associated with severe COVID-19 illness but can also present in people with COVID-19 who were physically asymptomatic or had mild physical symptoms of COVID-19. In our population, delirium was associated with a range of variables, including hypoxia, older age, elevated CRP, and a prolonged hospital length of stay compared to patients with other psychiatric diagnoses. In addition, delirium was associated with significant mortality (17%). This is consistent with the findings of other studies, which show that delirium is associated with poor outcomes, including mortality, in COVID-19 patients^23^. Thus, the early recognition of delirium may improve the management and outcomes of a subgroup of COVID-19 patients at high risk of poor outcomes.

Furthermore, clinicians caring for COVID-19 patients should be aware of the risks associated with delirium. The early identification and management of delirium should be integral to COVID-19 clinical protocols. We hope that this study may assist with resource allocation and the planning of CL services. Further studies are needed to investigate the longer-term psychiatric sequelae of COVID-19.

## Declarations

### Ethical approval and consent to participate

This study was approved by the Institutional Review Board of Hamad Medical Corporation (MRC-05–072).

### Availability of data and materials

The data that support the findings of this study are available from the corresponding author upon reasonable request and pending additional ethical approval.

### Competing interests

Competing interests: PMH reports personal fees from Janssen, NewBridge Pharmaceuticals, and Otsuka, outside the submitted work.

### Funding

This study did not receive any specific grant from funding agencies in the public, commercial, or non-profit sectors.

### Acknowledgments

We are thankful to Prem Chandra who provided statistical analysis assistance to the research team.

## Figures and Tables

**Table 1:** Sociodemographic and clinical details of 100 consecutive SARS-CoV-2 positive patients referred to the HMC consultation–liaison psychiatry service.

Characteristic	Number (%)
Age (years) Median Mean Range Male	43 45.92 18-91 92 (92%)
Nationality South Asian (Indian, Pakistani, Bangladeshi, Nepalese, Afghan) Qatari Other Arab Filipino Caucasian African	60 (60%) 18 (18%) 14 (14%) 4 (4%) 2 (2%) 2 (2%)
Source of psychiatric referral Ward Emergency Department	79 (79%) 21 (21%)
Length of hospital stay (days) Median Mean Range	20 26.51 1-155
Physical comorbidity present	67 (67%)
Includes Cardiovascular Disease, Diabetes, Liver Disease, Lung Disease, Cancer, Chronic kidney disease	
Severity of COVID-19 infection^1^ Asymptomatic Mild COVID-19 Mild COVID-19 pneumonia Severe COVID-19 pneumonia Critical	27 (27%) 17 (17%) 21 (21%) 21 (21%) 14 (14%)
Details of COVID-19 treatment	
Supplementary oxygen	50 (50%)
Steroids	45 (45%)
Hydroxychloroquine	50 (50%)
Antibiotics/antivirals	79 (79%)
Past psychiatric history	35 (35%)
Maintenance psychotropic medications prescribed at the time of admission	17 (17%)
Significant psychosocial stress before admission	48 (48%)
Psychiatric diagnosis made by the CL team^2^ Delirium Acute stress reaction/adjustment disorder Depression (includes 1 case of depressive psychosis) Mania Anxiety disorders Non-affective psychosis Dementia (all cases present pre-COVID-19) No psychiatric diagnosis made Miscellaneous (stroke, intellectual disability with challenging behavior, cerebral palsy, epilepsy, metabolic encephalopathy, Herpes Simplex encephalitis, alcohol withdrawal)	29 (29%) 24 (24%) 16 (16%) 15 (15%) 14 (14%) 13 (13%) 6 (6%) 1 (1%) 10 (10%)
Psychotropic medication prescribed during admission	85 (85%)
Psychiatric condition resolved or improved at the time of hospital discharge	87 (87%)

^1^Severity of COVID-19 infection related to the most severe illness during the hospital admission and was made by the medical team as per the HMC treatment protocol as below. (a) asymptomatic: i.e., no characteristic physical symptoms of COVID-19 infection (b) mild COVID-19: uncomplicated upper respiratory tract viral infection, may have non-specific symptoms, such as fever, cough, sore throat, nasal congestion, malaise, headache, or muscle pain; elderly people and individuals who are immunosuppressed may present with atypical symptoms (c) mild pneumonia: patients with pneumonia and no signs of severe pneumonia (d) severe pneumonia: fever or suspected respiratory infection, plus one of the following: (i) respiratory rate 1>30 breaths/min, (ii) severe respiratory distress, or (iii) SpO2 <90% on room air (e) critical disease: acute respiratory distress syndrome, sepsis, septic shock ^2^The sum of psychiatry diagnoses exceeds 100, as some patients had more than one psychiatric diagnosis.

**Table 2:** Comparison of the sociodemographic and clinical characteristics of patients with and without delirium

Characteristic	Clinical Diagnosis n (%) or Mean±SD		*P* value (2-tailed)	
	*Delirium (n=29)*	*No delirium (n=71)*	
Age	59.45 (17.49	40.39 (13.25	0.000
Gender			
Male	28 (30.4)	64 (69.6)	0.430
Female	1 (12.5)	7 (87.5)	
Ethnicity			
Qatari	2 (11.1)	16 (88.9)	0.119
Non-Qatari	27 (32.9)	55 (67.1)	
Referral source			
Emergency department	2 (9.5)	19 (90.5)	0.052
Inpatient units	27 (34.2)	52 (65.8)	
Physical comorbidity			
Yes	24 (35.8)	43 (64.2)	0.056
No	5 (15.2)	28 (84.8)	
Current smoker			
Yes	2 (13.3)	13 (86.7)	0.349
No	26 (31.7)	56 (68.3)	
Current alcohol or substance use			
Yes	1 (16.7)	5 (83.3)	0.669
No	28 (29.8)	66 (70.2)	
Past psychiatric history			
Yes	7 (20.0)	28 (80.0)	0.222
No	22 (33.8)	43 (66.2)	
Maintenance psychotropics medication on admission			
Yes	1 (5.9)	16 (94.1)	0.020
No	28 (33.7)	55 (66.3)	
Psychosocial stress			
Yes	6 (12.5)	42 (87.5)	0.001
No	23 (44.2)	29 (55.8)	
Severity of COVID-19			
Non-severe disease	11 (16.9)	54 (83.1)	0.001
Severe disease	18 (51.4)	17 (48.6)	
Oxygen administered			
Yes	25 (50.0)	25 (50.0)	0.000
No	4 (8.0)	46 (92)	
Treatment with antibiotics/antivirals			
Yes	28 (35.4)	51 (64.6)	0.013
No	1 (4.8)	20 (95.2)	
Treatment with chloroquine/hydroxychloroquine			
Yes	18 (36.0)	32 (64.0)	0.019
No	11 (22.0)	39(78.0)	
Treatment with steroids			
Yes	21 (46.7)	24 (53.3)	0.001
No	8 (14.5)	47 (85.5)	
Psychotropic medication prescribed during index admission			
Yes	26 (30.6)	59 (69.4)	0.543
No	3 (20.0)	12 (80.0)	
Length of hospital stay	38.48 (36.71)	21.62 (15.10)	0.001
Lowest oxygen saturation during index admission	84.10 (15.59)	93.84 (5.16)	0.000
Highest C-reactive protein value during index admission(Normal range=0–5 mg/L)	227.36 (122.34)	122.82 (135.46)	0.001
Lowest lymphocytes count during index admission(Normal range=1–3×10^3^ /microliter)	1.11 (1.27)	1.60 (0.91)	0.033
Highest Neutrophils count during index admission(Normal range=2–7×10^3^ /microliter)	6.77 (4.26)	6.14 (5.26)	0.569

**Table 3:** Binary logistic regression using a stepwise method to look for independent factors associated with delirium (variables that were significant in the univariate analysis were entered into the analysis). Only significant predictors are shown in the table.

Predictor variables			95% CI for EXP(B)		
		Exp(B)	Lower	Upper	*P*-value
Step 1	Age	15.60	3.36	72.26	0.000
Step 2	Age	63.28	3.95	1013.87	0.003
	Lowest oxygen saturation during index admission	0.835	0.74	0.934	0.002
